# Paramedics and Physician Assistants in Israel

**DOI:** 10.1186/s13584-019-0358-9

**Published:** 2020-01-06

**Authors:** Roderick S. Hooker

**Affiliations:** Israel Journal of Health Policy Research, 115917 NE Union Rd, Unit 45, Ridgefield, WA 98642-8706 USA

**Keywords:** Trauma, Burnout, Task transfer, Physician associates

## Abstract

Israeli emergency medicine is undergoing change. The paramedic is experiencing high separation rates because the position is understaffed, overworked, and underpaid. Physician assistants (PAs) were introduced into the emergency department by training paramedics and to date they seem satisfied with this new role. Experience in other countries indicates that PAs can improve access to care, reduce errors, increase efficiency and have satisfying roles in health systems. The Israeli health system will need to determine if additional roles for PAs will be accepted by the public and physicians alike.

## Introduction

**Fig. 1:**
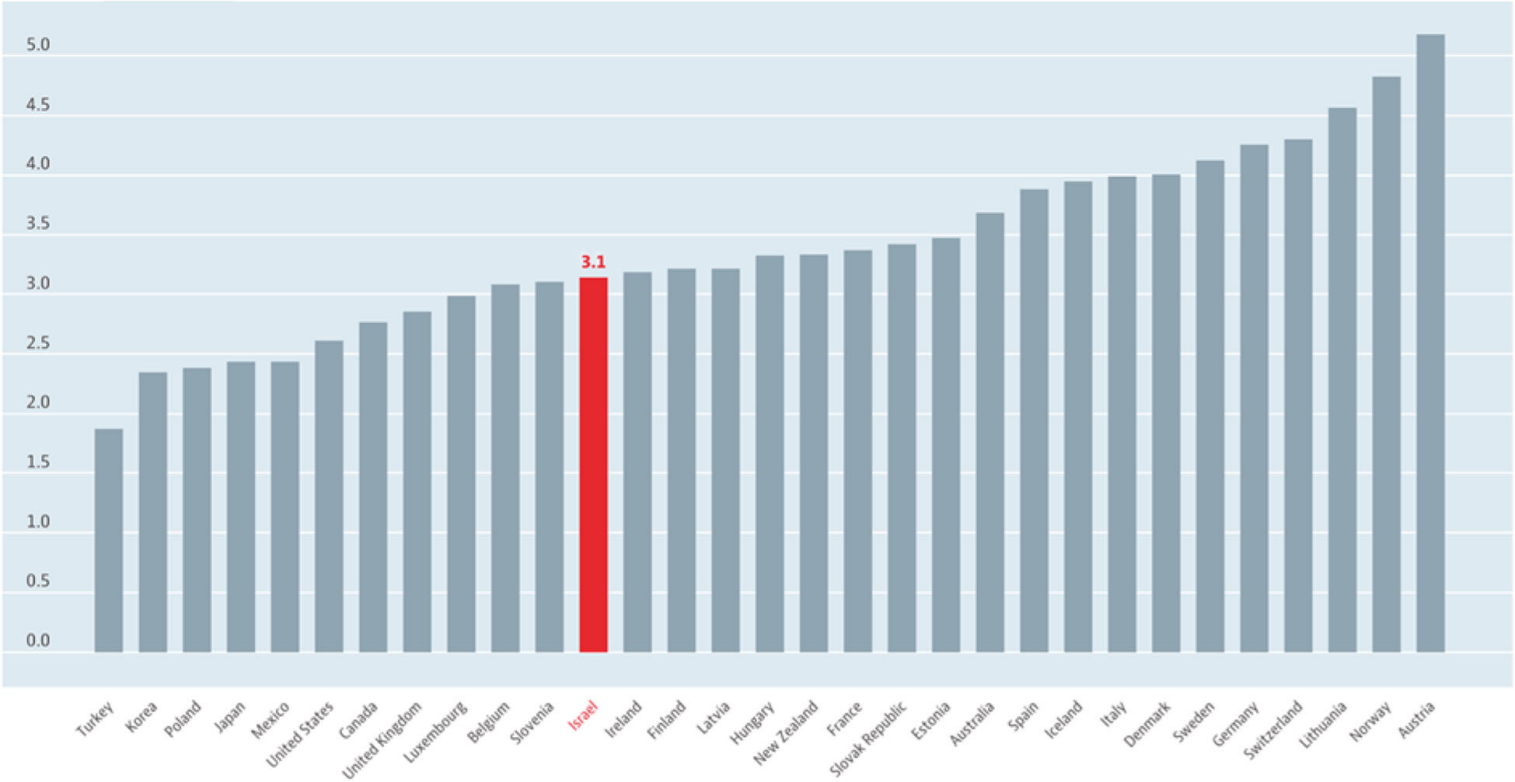
Israel had 3.1 doctors per 1,000 population in 2018

As of 2018, Israel had 3.1 clinically active physicians per capita, close to the median (3.2) of other countries highlighted in a report from the OECD [Organization for Economic Cooperation and Development] [1]. The availability of clinically active physicians in Israel is about 20% higher than in the U.S. (Fig. 1).

A number of attributes are notable about Israel: fiscal performance is rated good, strong macroeconomic growth is underway, unemployment is low, and its citizens enjoy universal healthcare [2]. Nonetheless, Israel is also struggling with some health professional issues and one, emergency medicine, is at the forefront. The challenge is the growing number of patients and an aging population without concurrent growth in staffing levels in emergency departments (EDs). Shortages in emergency medical staff have led to long waiting times, burnout, dissatisfied patients, and inefficient treatment [3].

In 2019 two papers were published in this journal about Israeli emergency medicine – with one focused on paramedics and the other focused on physician assistants (PAs) [3, 4]. Dopelt et al. described Israeli paramedics as spread thinly, about 8 per 100,000, when compared to the US with approximately 25 per 100,000 (https://www.bls.gov/oes/current/oes292041.htm). They report that 73% depart after 5 years and 93% have left their paramedic role after 10 years – a high rate of turnover compared with most other health professions. Various reasons are offered for the Israeli paramedics’ departure. “I felt that I had no career advancement opportunities” ranked high on the list – 83%.

Maoz-Breuer and colleagues discuss the introduction of PAs into Israeli EDs. Two graduation cohorts of 30 each have been deployed in 17 of the 34 EDs around the country. All of the graduates were paramedics, and all were part of a pilot study undertaken by the Ministry of Health to see if an unsatisfactory phase in the career of a paramedic can serve as a springboard for a more sustaining one. In the Maoz-Breuer et al. paper, the survey of the ‘former-paramedics-now-PAs’ reported personal fulfillment, career prospects, and wage improvement as leading reasons for job or role satisfaction.

## Discussion

Introducing PAs into a healthcare system is not new. As of this writing there are 19 countries with some form of PA and many others with comparable health professionals such as assistant medical officers, clinical officer, or feldshers [5, 6]. How they are defined and the roles they provide are constantly changing as new technology alters the healthcare landscape, populations age and grow, and policy is adjusted to accommodate supply and demand.

PAs and their counterparts offer flexibility in adaptation and deployment in diverse settings. This is facilitated by the educational system, since adjusting a PA program’s didactic and clinical syllabi is easier than changing the curriculum of a medical school. Attitudes also change. Early on in America, resistance to introducing PAs was felt in some sectors of society and a few medical specialties [7]. However, initial recruiting efforts relied on former corpsmen and medics, most having honed their skills during the Viet Nam war era, and that background seemed to resonate with citizens [8]. Patients’ confidence grew and, after a few decades, the PAs became a recognized and respected group of health professionals [9]. Satisfaction came along quickly. Patients were willing to see any reliable health provider as long as their needs were met, and quality was not compromised [10].

One compelling reason to employ a worker who can be entrusted with a broad range of medical tasks, such as trauma care, is experience in the military where such care has been the realm of health professionals such as the surgeon, nurse, and medic working as a team. The Israeli Defense Forces Medical Corps is legendary not only for its training but for extending humanitarian aid where needed. The medic plays a particular role in this service [11]. The paramedic is the civilian counterpart, but, as the Dopelt group points out, the role is no longer satisfying over the long-term.

The PA in Israel is the newest member of a growing cadre of health professionals, and with it are opportunities to build upon the successes and failures of other nations [12]. Utilizing paramedics as PAs and employing them in EDs seems to make sense and seems to be working. The next step is to scale up the PA project to see if their adaptability can extend beyond the ED setting and into other medical venues. This will require examining the universal use of PAs, canvassing societal need, and modifying their roles to fit the needs of Israeli society.

PA roles across the globe vary widely and are expanding in ways unpredicted. One example includes facilitating joint replacement where the annual productivity of a Manitoba orthopaedic team increased by 42% just by adding a PA for pre- and post-operative patient assessment [13]. Management of complex diseases in US veterans by PAs produces outcomes indistinguishable from physicians but at a lower labor cost [14]. In the Netherlands, the hospital-based PA is taking on procedures normally reserved for medical and surgical specialized doctors, freeing them up for more complex tasks [15]. The New Zealand ED PAs were not only able to integrate smoothly with the existing team, but supervising physicians reported they added value by “the quality and safety of the work performed” [16]. In the UK the issues facing the British in accessing adequate healthcare led to the decision that they needed a large cadre of PAs. In the UK, PAs are called Physician Associates. Not only did the NHS fund 25 additional PA programs in the second decade but it incorporated them in The Royal College of Physicians [17].

One of the characteristics of the PA concept is the relationship between the PAs and physicians. In Ireland the definition of a PA is: “a healthcare professional trained in medicine who works as part of a medical team in partnership with doctors to provide medical care to patients” [18]. Outside of the military, the PA is generally not autonomous and instead defines him or herself as a team member – with the physician central to the group. This collaborative relationship seems to not only delineate their nature and role but appears to be the adaptation needed to the ever-changing dynamics of contemporary medicine. This physician-PA relationship is true for the Israeli PA in the same way it is for the PA in Australia, Germany, or any other location.

If Israel is to welcome PAs, the health organization pragmatist will want to know the evidence pertinent to their inclusion. For example, the willingness of patients to be seen by a PA needs to be assessed along with patients’ satisfaction at having had their needs met. Other questions are a bit more challenging; for example, can a country’s physician corps embrace the collaborative type of practice that PAs bring? Is a hierarchical system of medical authority open to the growing trend in team-based care when that team member is a PA? Will the phenomenon of growing physician burnout, which is emerging in a number of countries, be ameliorated as it has in sectors where PAs work in partnership with physicians [19]? If being a PA is career satisfying, will that have a carryover effect on their supervising physician [20]?

## Conclusion

Israel is at the forefront of many medical and technological advances; and introducing PAs into a mature healthcare system, should be a very good addition to Israel’s medical workforce. The early results in emergency medicine seem promising. The challenge will be extending this success, seeing if more paramedics can be attracted to becoming PAs rather than leaving healthcare entirely, and determining if the medical system and general public will embrace PA roles not only in the ED but in other fields as well.

## Abbreviations

EDs: Emergency departments
OECD: Organization for economic cooperation and development
PA: Physician assistant or physician associate
U.S.: United States
UK: United Kingdom
